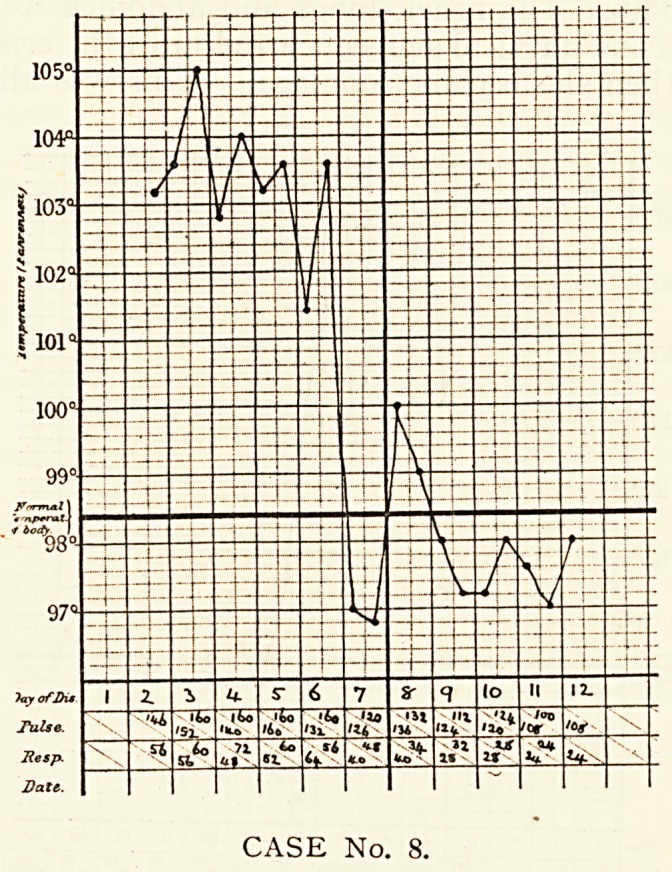# On the Cerebral Symptoms of Lobar Pneumonia in Children

**Published:** 1913-12

**Authors:** F. H. Edgeworth

**Affiliations:** Physician to the Bristol Royal Infirmary and Professor of Medicine, University of Bristol.


### ON THE CEREBRAL SYMPTOMS OF LOBAR PNEUMONIA IN CHILDREN.

BY

F. H. EDGEWORTH, M.D., D.Sc.,

*Physician to the Bristol Royal Infirmary and Professor of Medicine, University of Bristol.*

---

It has long been known that children suffering from pneumonia may show signs of disturbance of the central nervous system which accompany those due to the pulmonary condition. Considerable difficulties in diagnosis may occur in such cases, and it is the object of this paper to indicate the means whereby, in some at least, a true opinion as to the nature of the illness may be arrived at.

Eight cases are recorded. They have been under observation at the Bristol Royal Infirmary during the last seven years. During the same length of time some sixty-three cases of lobar pneumonia in children were admitted to my wards, so that it would appear that about one case in eight shows signs of cerebral disturbance.

In the short histories here given the actual dates of admission are omitted, and the dates of appearance of the phenomena are stated in relation to the illness. The temperature charts are so constructed that the first day of the disease occupies the first left-hand column, irrespective of the day of admission. The abrupt onset, so characteristic of lobar pneumonia, permits of this being done with fair accuracy. The varying duration of the fever and the onset of pulmonary physical signs (marked by a cross) can thus be seen at a glance.

**Case 1.**—Freddie R., aged 11, was admitted on the second day of his illness. On the previous day he had come home from school with headache, pain in the stomach and vomiting, and grew worse, continuing to vomit, and having incontinence of urine and fæces. On admission he was found to be in a semi-comatose condition, lying curled up in bed and resenting being touched, with marked retraction of the head. The tongue was furred and dry. No abnormal physical signs were present in chest and abdomen. The knee-jerks were present, but not exaggerated. Kernig's sign was present. On the third day it was found that the breath sounds over the upper part of the

CASE No. 1.

right lung were harsh in character, and on the fifth day the upper part of the right side of the chest was dull on percussion, with bronchial breathing and a few crepitations. Kernig's sign was still present. On the eighth day the legs could be raised to an angle of 60° with the trunk, on the twelfth to an angle of 75°, and on the sixteenth to a right angle. Meanwhile—on the tenth day—a crisis in the temperature took place with great improvement in the general condition. Convalescence was rapid.

**Case 2.**—Marion H., aged 8 years, was admitted on the fourth day of the disease. During the previous three days she had suffered from headache and repeated vomiting. She was found to have a temperature of 105.2°, a weak, regular pulse of 128, and a respiration rate of 48. She had a hot, dry skin and flushed face. No inequality of chest movement could be detected and no abnormality was found on examination. The spleen was just palpable, the liver not enlarged, and the abdomen normal. The knee-jerks were obtainable on reinforcement. The plantar reflexes were flexor in type. On the fifth day physical signs of pneumonia were found in the right upper lobe—dulness on

CASE No. 2.

percussion, increased vocal fremitus and resonance, bronchial breathing, and a pleural friction sound in the axilla. Herpes had developed on the left side of the mouth. On the sixth day a crisis occurred, and the patient soon recovered.

**Case 3.**—Gilbert J., aged 11, was admitted on the first day of his illness. He had been quite well until that morning, when he complained of frontal headache, thirst and feeling hot. He did not shiver or vomit. On admission he was found to be a well developed boy, lying in a semi-comatose state, though crying out and wriggling on being examined. The pupils could not be tested with accuracy owing to the patient's movements. The pharynx and tonsils were very red. No abnormality could be detected in chest or abdomen. The knee jerks were absent, an extensor plantar reflex in both feet, and Kernig's sign on the left side only. Incontinence of urine. A throat swab was taken, and showed the presence of Hoffman's bacillus and staphylococci. The cerebro-spinal fluid was clear, not under pressure and contained no cells or micro-organisms. On the second day

CASE No. 3.

no change had taken place. On the third day there was dulness on percussion at the right apex down to the third rib, with distinct tubular breathing and pleural friction, but no crepitations. The general state had improved, and the patient was fully conscious. On the seventh day a crisis occurred, and the patient rapidly got well.

**Case 4.**—Daniel M., aged 3, was admitted on the second day of his illness. On the evening of the previous day convulsions had occurred, and there had been another attack on the morning of admission. He was found to have a temperature of 102.4°, a pulse rate of 156, and respiration of 56. At the base of the left lung there was dulness on percussion, high-pitched breath sounds and a few crepitations. The heart and abdomen were normal. He had a third fit about one hour after admission, but no others. A fall of temperature began on the sixth day, and

CASE No. 4.

was continued on the seventh—down to 97.8°. The boy quickly recovered.

**Case 5.**—Ernest G., aged 4, was admitted on the first day of his illness. He had had convulsions, though without any fever, on two previous occasions, and had been an in-patient sixteen and twenty-two months respectively prior to this attack. He was seized with convulsions about four hours before admission, and these had continued. He was found to be in a semi-conscious condition, with general twitchings occurring frequently. There was no paralysis of face, arms or legs. The temperature was 101°, pulse 144, very small and weak, and respiration 44. No abnormal physical signs were found in chest or abdomen. The pupils were equal, of medium size and reacted to light, and the corneal reflexes were present. Both knee-jerks were present and exaggerated; both plantar reflexes extensor in type, and Kernig's sign was present. The patient was put into a hot bath and given ol. ricini, followed by potass. bromidi and chloral hydrate. Three hours later he was found to be asleep. A nose swab was taken; this was reported to show Hoffman's, but no Klebs-Lœffler's bacilli. On the second day the convulsions had ceased, and the boy had slept well. The ocular fundi were found to be normal. On the fourth day the temperature was still high. A few crepitations and tubular breathing, with dulness on percussion, were found at the right apex. Slight head retraction was present, and pain was complained of on pushing the head forwards. Kernig's sign was still present. The plantar reflexes had become flexor in type. On the sixth day the boy was better and did not cry when touched; the head retraction had disappeared. Kernig's sign was not so definite. Lumbar puncture showed the presence of clear

CASE No. 5.

cerebro-spinal fluid, not under pressure, and containing no organisms. The upper lobe of the right lung was in the same condition as on the fourth day. On the eighth day Kernig's sign was absent. On the tenth day a crisis occurred and the patient seemed much better. He was sent out well a fortnight later.

**Case 6.**—Lily M., aged 10, was admitted on the second day of her illness. On the previous day, when apparently in good health, she suddenly fell down in a fit, and since then had been in an unconscious condition, occasionally breaking out into a shrill cry. On admission, the girl was restless, unconscious, shrieking when disturbed. She lay flat on her back in bed. The face was red, but not cyanosed, with wide-opened mouth and closed eyelids. The pupils were equal, of medium size, and did not react to light; the conjunctival reflexes were present, though sluggish. No abnormal physical signs were found in chest and abdomen. The knee-jerks were present, the plantar reflexes absent, and Kernig's sign was present.

On the third day Kernig's sign had disappeared, and the plantar reflexes were still absent. Nothing abnormal was found on examination of the lungs. Spinal puncture showed the presence of a clear cerebro-spinal fluid, not under pressure, and sterile on culture. On the fourth day a crisis in the temperature

CASE No. 6.

occurred. On the fifth day the patient became partially conscious. Until this date there had been incontinence of urine and fæces. On this day, for the first time, a patch of dulness, the size of half a crown, was found at the base of the left lung, and in this area crepitations could be heard. On the seventh day consciousness was fully regained. Convalescence was uneventful.

At no time did the ratio of respiration to pulse rise above 1.3.

**Case 7.**—Dora M., aged 14, was admitted on the fourth day of her illness, which had begun suddenly with considerable pain in chest, abdomen and legs, vomiting, and a feeling of great weakness. The vomiting had been incessant during the first three days ; no rigor had occurred. She lay in bed in dorsal position, with flushed, anxious face, and rapid breathing. There was a herpetic eruption on the upper and the left side of the lower lip. The heart, lungs and abdomen were normal. No change in the physical signs occurred until the crisis. Every night until then she became maniacal. This was allayed by a

CASE No. 7.

hypodermic injection of morphia and strychnine. She vomited from time to time. On the eighth day a sudden fall in the temperature took place, and on this day, for the first time, fine crackling inspiratory crepitations with prolonged high-pitched expiration were heard at the base of the right lung. Convalescence was rapid.

**Case 8.**—Edward M., aged $1\frac{3}{12}$, was admitted on the second day of his illness. On the previous one he had begun to suffer from vomiting and diarrhœa. He was found to be a well-nourished boy, with a temperature of 103.2 deg., pulse rate 146, and respiration rate 56. The head was markedly retracted, and the whole back slightly arched, with rigidity of the muscles ; the pupils were equal and widely dilated. The child was restless and cried on being moved. No abnormal physical signs were found in chest or abdomen. This condition persisted until the seventh day, when a crisis in the temperature occurred, and the child improved. At no time were any abnormal physical signs found in the chest. After the crisis the head

CASE No. 8.

retraction and opisthotonus diminished, but were still present in less degree on the eighth day, and then disappeared. Convalescence was uneventful.

On comparison of these cases it appears that in all the fever, or at least the illness, set in abruptly, and in seven out of the eight cases fell by crisis after lasting from three to nine days. The date of appearance of abnormal physical signs in the chest was variable, from the third to the eighth day of the illness; but in six of the eight cases the normal respiratory-cardiac ratio of 1.4½ or 1.4 was changed to 1.3 or 1.2½, and this, taken in association with the history of abrupt onset and hot, dry skin, permitted an approximate diganosis of the pulmonary condition before the appearance of physical signs in the chest. In one case (7) the ratio was not altered, probably owing to the small area of lung affected, and doubt as to the cause of the fever persisted until the eighth day, when, coincident with the crisis, a small patch of dulness and crepitation was found at the base of the right lung. In another case (8) no physical signs of pneumonia were ever discovered, but the respiratory-cardiac ratio varied between $1.2\frac{1}{2}$ and $1.2\frac{1}{5}$, and the temperature dropped by crisis on the seventh day.

The symptoms of cerebral irritation which these cases presented may conveniently, though not quite accurately, be divided into those with which the illness began and those which accompanied the major portion or the whole duration of the pyrexial period.

The initial symptoms were headache, convulsions and vomiting. Headache and vomiting occurred either alone or in combination, whereas convulsions (as in cases 4, 5, 6) were not associated with either headache or vomiting. Headache and convulsions were transitory, and did not continue beyond the second day, whereas vomiting (as in case 7) might continue during the whole of the fever. This fact and its occasional association with diarrhœa suggest that, at least in some cases, it may be due to a direct toxic influence on the gastric mucous membrane and not the result of cerebral irritation. In no case did a rigor occur, as is so often the case in adults.

The more abiding symptoms of cerebral irritation were coma, mania, retraction of the head, Kernig's sign, and an extensor plantar reflex. In no case did any of these symptoms develop after admission to the Infirmary; they were present when the child was first seen, *i.e.* at dates varying from the first to the fourth day of the illness. This fact is of importance in the diagnosis, for when pneumococcal meningitis occurs as a complication of pneumonia the symptoms usually begin late in the illness. This complication is very rare, and I have not seen it in cases under twelve years of age.

Coma occurred in three cases, with or without head retraction, but in each case with Kernig's sign; mania in one case; head retraction in three cases, with or without coma, and in two cases with Kernig's sign.

## Figures and Tables

**CASE No. 1. f1:**
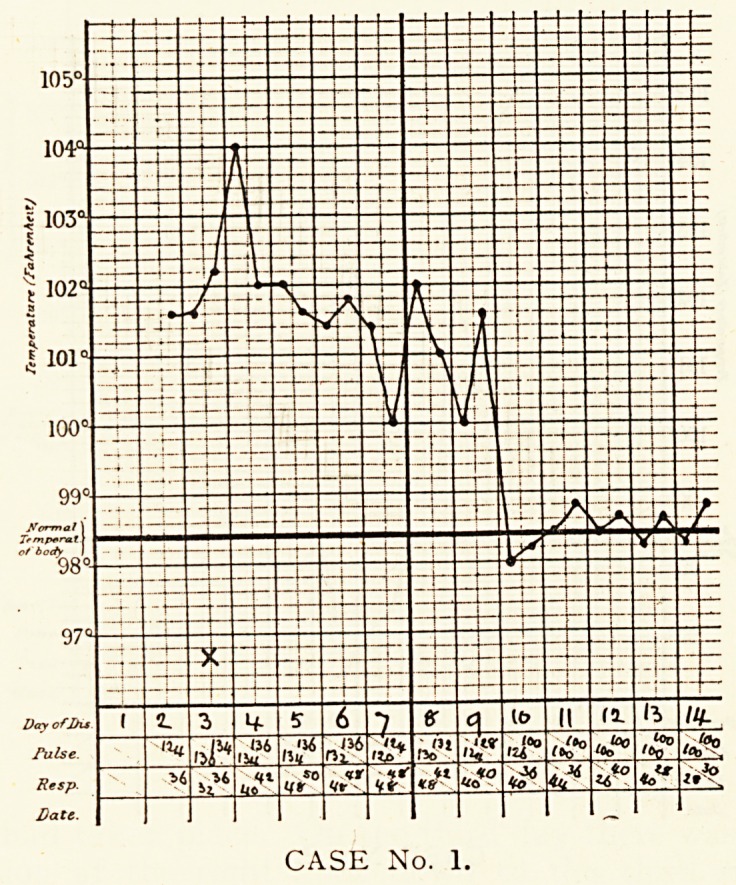


**CASE No. 2. f2:**
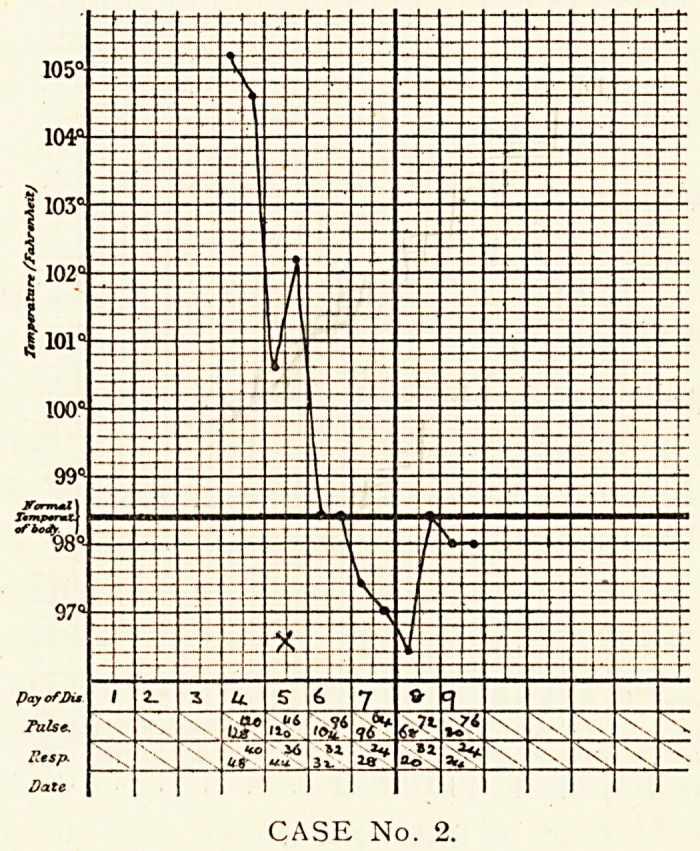


**CASE No. 3. f3:**
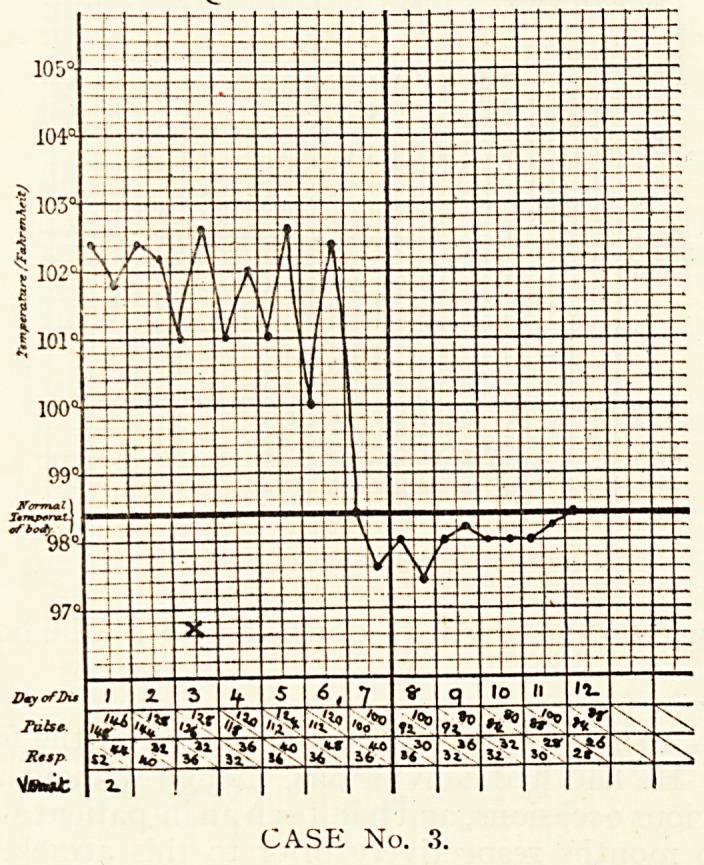


**CASE No. 4. f4:**
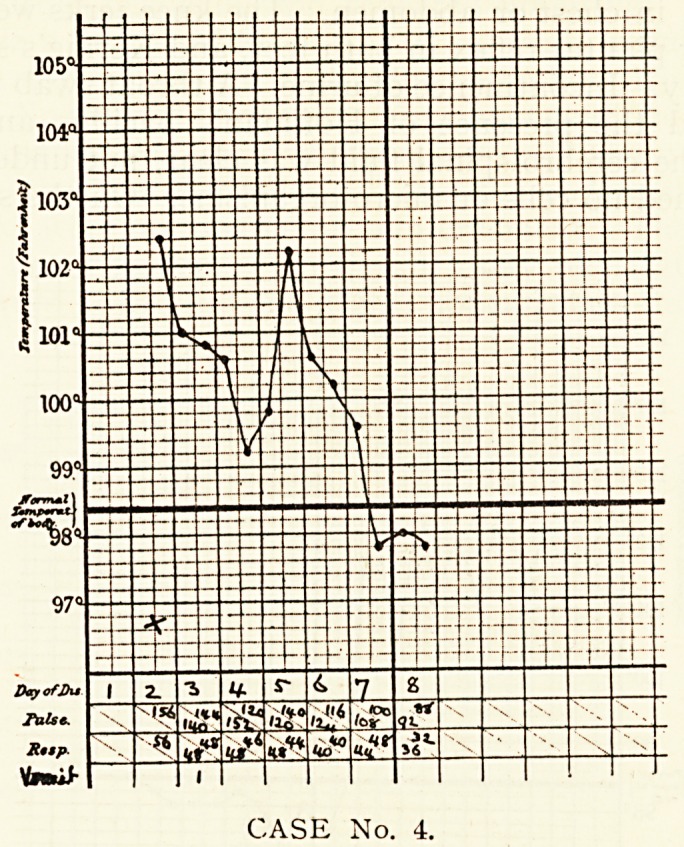


**CASE No. 5. f5:**
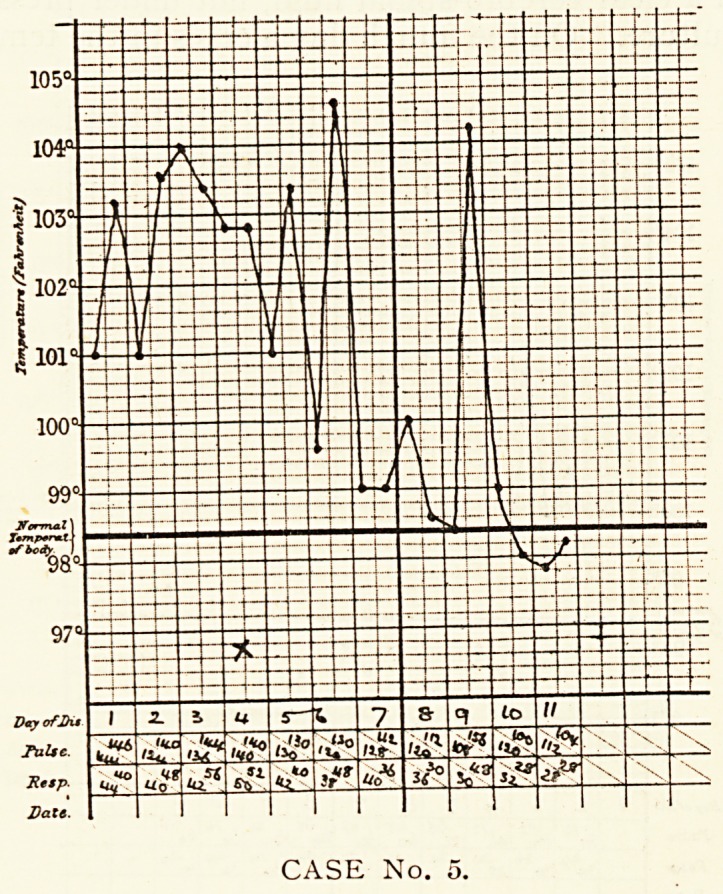


**CASE No. 6. f6:**
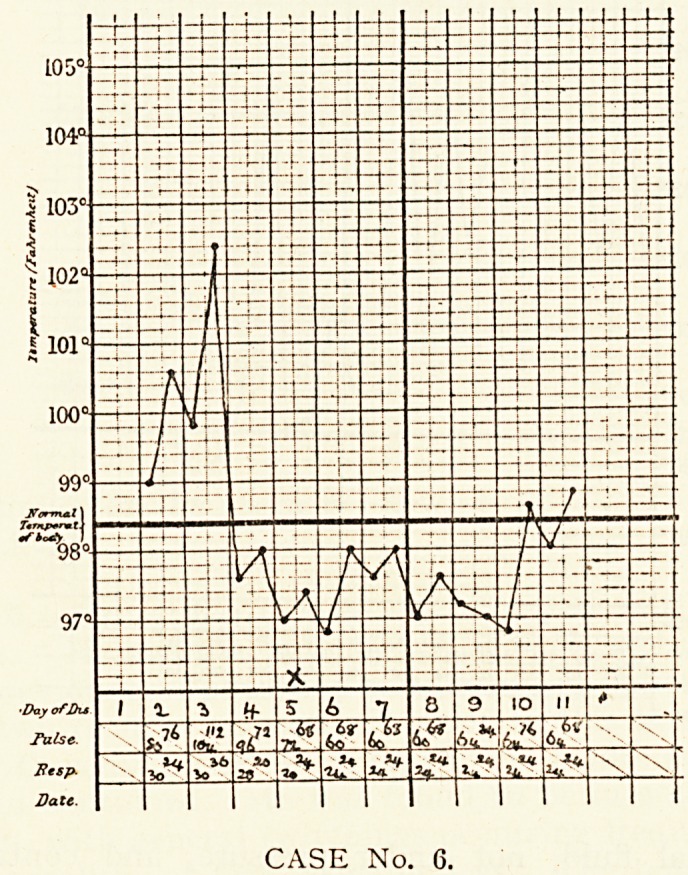


**CASE No. 7. f7:**
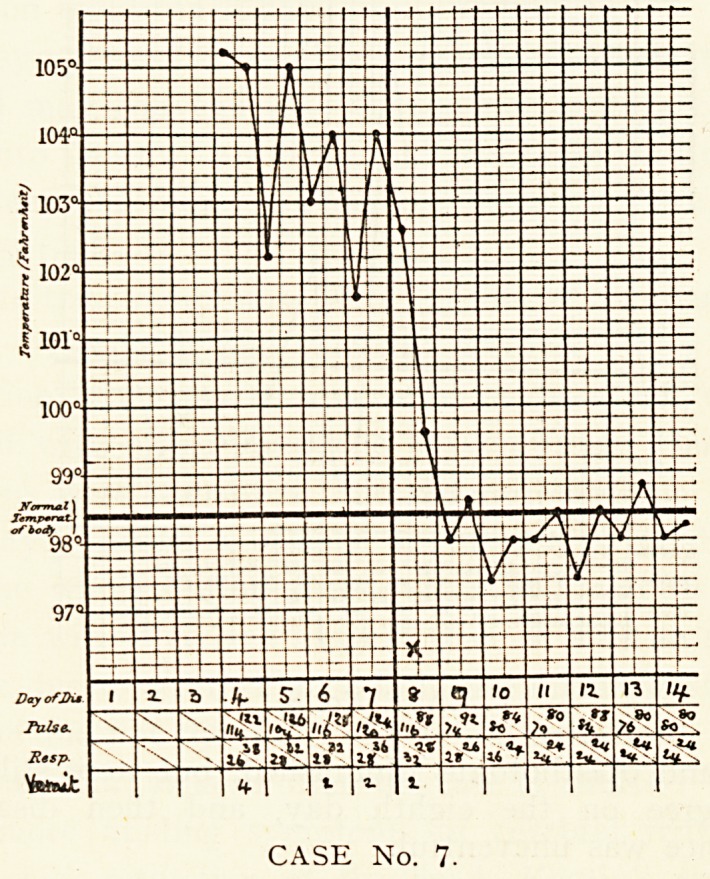


**CASE No. 8. f8:**